# Evaluation of autoimmune liver disease natural history in patients referred to Middle East Liver Diseases (MELD) center

**DOI:** 10.1186/s12876-023-03105-7

**Published:** 2024-01-04

**Authors:** Seyed Erfan Mehdi Nejad, Mohammad Heiat, Mohammad Javanbakht, Seyed Moayed Alavian, Mohammad Ali Abyazi Haris

**Affiliations:** 1 Baqiyatallah Research Center for Gastroenterology and Liver Diseases (BRCGL), Clinical Sciences Institute, Baqiyatallah University of Medical Sciences, Tehran, Iran; 2 Nephrology and Urology Research Center, Clinical Sciences Institute, Baqiyatallah University of Medical Sciences, Tehran, Iran; 3 Middle East Liver Diseases (MELD) Center, Tehran, Iran

**Keywords:** Autoimmune hepatitis (AIH), Autoimmune liver disease (AILD), Natural history, PBC, PSC

## Abstract

**Background:**

Autoimmune liver diseases (AILD) are increasing and common forms of chronic liver disease (CLD) with different clinical responses and characteristics which can result in cirrhosis. This study aimed to investigate the natural history and characteristics of AILD in an Iranian population.

**Methods:**

Patients with AILD [Autoimmune Hepatitis (AIH), Primary Biliary Cholangitis (PBC), Primary Sclerosing Cholangitis (PSC) and Overlap Syndrome (OS)] referred to Middle East Liver Diseases (MELD) center, Tehran, Iran, between January 2002 and December 2022 were included in this retrospective cohort study. The main features of natural history (the trends of liver functional tests (LFT), Auto-Antibodies, response to treatment and cirrhotic status) along with demographic data were studied.

**Results:**

Two hundred sixty-five patients (160 (60.4%) AIH, 37 (14.0%) PBC, 20 (7.5%) PSC, 48 (18.1%) overlap syndrome) with a median follow-up time of 5 years (IQR 4 to 8 years) were included. Baseline laboratory tests revealed that patients with AIH exhibit elevated transaminase levels. However, patients suffering from PBC and PSC displayed increased alkaline phosphatase levels. Conversely, in overlap syndrome patients, both transaminases and alkaline phosphatase were observed at high levels. Autoantibodies represented themselves as important diagnostic markers for the AIH and PBC but not for PSC. The complete response occurred in 112 (70%) of and 28 (58.4%) patients with AIH and overlap syndrome respectively and 21 patients 11 (6.9%) of AIH and 10 (20.8%) of overlap syndrome) were non-responders. Other patients in these two categories were considered as insufficient responders. On the other side, 32 (91.9%) and 8 (40%) of patients with PBC and PSC biochemically responded to Ursodeoxycholic Acid (UDCA). Unpredictably, cirrhosis regression was observed in some AIH and PBC patients.

**Conclusion:**

Appropriate medication management for AILD patients may leads to regression from cirrhosis and improvement of manifestations; while discontinuation of medication may cause relapses. However, patient suffering from PSC showed limited response to treatment.

## Background

Autoimmune liver disease (AILD) is a common form of chronic liver disease (CLD) consisting of autoimmune hepatitis (AIH), primary biliary cirrhosis (PBC), and primary sclerosing cholangitis (PSC) as three major entities and [1] a fourth yet rare entity of IgG4-related cholangiopathy [2]. In AIH, hepatocytes are primarily affected, while PBC and PSC affect small interlobular and medium size intrahepatic and extrahepatic bile ducts, respectively [1, 3]. In some cases, overlap syndrome (OS) has been described when concomitant manifestations of PBC or PSC are present alongside AIH [2]. Moreover, PBC-PSC overlap syndrome has been reported in the literature; however, it is very rare.

Epidemiologic studies conducted in northern America and Europe revealed an increasing trend from 1987 to 1994 for PBC, while a steady prevalence has been reported in recent studies. Recent studies suggest an increasing trend regarding AILD prevalence in the Asian region with Japan reporting an approximate 2.5 folds increase in AIH prevalence from 8.7 to 23.9 cases per 100,000 people from 2004 to 2016 [2]. The prevalence of PBC is 1.91 to 40.2 cases per 100,000 with an incidence rate of 0.03 to 0.58 cases per 100,000 globally [4]; studies also reported an incidence rate and a prevalence rate of 1.3 and 16.2 cases per 100,000 for PSC, respectively [2].

AILD treatment aims at relieving the symptoms, and to control hepatic inflammation to achieve biochemical responses [5]. The current treatment strategy for AIH, and PBC and PSC consists of immunomodulators, glucocorticoid therapy and ursodeoxycholic acid (UDCA) administration [6]; however, clinical response and disease characteristics differ among patients, and complete biochemical response are not achieved in some cases.

AILD, as a result, in spite of not being clear etiologically, can result in acute decompensation, liver failure, and cirrhosis, and can cause significant morbidity and mortality as a CLD [7, 8].

Due to the rising trend of AILD [9] and its significance in causing end-stage morbidity and mortality [10], this study aims at assessing the natural history and characteristics of AILD in an Iranian population. A limited number of studies have investigated the characteristics of autoimmune liver disease (AILD) and its natural history (the trends of liver functional tests (LFT), Auto-Antibodies, response to treatment and cirrhotic status) in response to treatment in the Middle East and North Africa.

## Materials and methods

Patients treated between January 2002 and December 2022 at the Middle East Liver Disease (MELD) center, Tehran, Iran were included in this retrospective cohort study. All electronic and paper medical records for patients with AIH, PBC, PSC, and overlap syndrome (OS) were researched. Patients were identified using the International Classification of Diseases (ICD-10), along with specific written diagnosis. Data were collected on demographics, clinical, serological, radiological, histological, and treatment variables. From a total of 320 individuals at baseline 55 with no clinical, biochemical, or histological data were excluded and finally 265 patients according to the simplified AIH diagnosis criteria published in 2008 by the International Autoimmune Hepatitis Group (IAIHG) [11]. The diagnostic accuracy of AIH simplified score, routinely used as diagnostic criteria, have been validated in some populations as well as Iranian [12, 13]. Patients diagnosed with AIH after the age of 11 is included, along with PBC, PSC, and overlap syndrome patients who meet the biochemical, autoantibody, liver histology, and cholangiography criteria. Autoantibody detection for ANA was done by solid-phase enzyme immunoassay (ELISA) (Pishtazteb.Co). ASMA, AMA, and Anti-LKM1, were assessed using indirect immunofluorescence assays (IFA), (AESKU.DIAGNOSTICS GmbH & Co. KG, Germany).

According to clinical presentations at the time of diagnosis, patients were classified as (1) Asymptomatic subjects who had abnormal biochemical tests, the presence of abnormal liver function tests, or laboratory positive test results. (2) Subjects who had non-specific symptoms including asthenia, anorexia, pruritus, fatigue, weight loss, and abdominal pain with evidence of abnormal biochemical tests of liver function (3) Subjects presented acute hepatitis with right upper quadrant pain, nausea, and jaundice, with a pattern of hepatocellular injury shown in the laboratory tests [14]. (4) Subjects with Cirrhosis of the liver, the presence of clinical (i.e., gynecomastia, telangiectasia, palmar erythema, collateral circulation, ascites, and encephalopathy in advanced levels), biochemical (hypoalbuminemia, thrombocytopenia, prolongation of prothrombin time), imaging signs of cirrhosis and pathologic evidence are also evaluated at baseline and during study follow-up time. The presence or absence of liver cirrhosis at presentation was defined according to the liver biopsies (35 cases out of 72 cirrhotic patients), clinical characteristics, and the results of ultrasonography and blood tests.

In response to treatment evaluation and study outcomes, AIH and overlap syndrome patients were classified as (1) non-responders if they did not manage a 50% decrease in serum transaminase levels within 4 weeks after initiation of treatment, using their starting point as a reference point. (2) Complete response if serum transaminase levels normalize within 6 months (3) Insufficient response if lack of complete biochemical response achieved and determined no later than 6 months [15]. Moreover, PBC syndrome patients’ response to treatment was evaluated and classified using biochemical response to UDCA treatment according to a decrease in ALP level > 40% of baseline level or normal levels after one year of UDCA treatment (Barcelona criteria) [16]. Although, there is no criteria for PSC response to treatment, regarding to other studies we considered biochemical parameters (majorly alkaline phosphatase [ALP] < 1.5 × upper limit of normal) and clinical features as response to treatment [17, 18].

Cirrhosis regression defined as decrease in child-pugh score or improvement in laboratory tests and clinical evidence and imaging findings (ultrasonography and fibroscan).

Data were collected by reviewing medical records in the hospital’s electronic registry using a previously designed data collection instrument. The investigators double-checked the medical records to ensure the quality of the data and reduce information bias. We followed up with the patients until 31 December 2022 or until the last documented date in the medical record. The continuous and categorical variables are presented as mean (standard deviation (SD)) and number (percentage), respectively. In the case of continuous variables that do not follow a normal distribution as determined by Kolmogorov-Smirnov’s test, the median (interquartile range) is shown. All statistical analyses were performed using SPSS statistical software, version 26. The sample size was not estimated a priori since all patients diagnosed with AILD and treated during the study period were included. Study protocols were approved by the ethical committee of the Research Ethic Committees of Baqiyatallah Hospital (Approval ID: IR.BMSU.BAQ.REC.1401.055), The final manuscript followed STROBE recommendations for the reporting of observational studies [19].

## Results

There were 265 patients who met the inclusion criteria. The median follow-up time was 5 years (IQR 4 to 8 years) with a range from 1 to 22 years. One hundred sixty (60.4%) of 278 patients had AIH, 37 (14.0%) had PBC, 20 (7.5%) had PSC, and 48 (18.1%) had overlap syndrome.

Their mean ± SD ages were 46.4 ± 14.0, 51.2 ± 12.7, 39.7 ± 9.5, and 43.1 ± 13.2 years, in AIH, PBC, PSC and overlap syndrome respectively. One Hundred Fourteen (71.2) in AIH, 31 (83.8) in PBC, 11 (55.0%) in PSC, and 32 (66.7) in overlap syndrome were women. Baseline mean ± SD BMI values for AIH, PBC, PSC, and overlap syndrome were 25.9 ± 3.9, 27.1 ± 4.2, 24.2 ± 3.9, and 24.1± 3.8 (Table 1).

**Table 1:** Baseline characteristics of study population

	AIH (n = 160)	PBC (n = 37)	PSC (n = 20)	Overlap syndrome (n = 48)
Age, yrs, mean (SD)	46.4 (14.0)	51.2 (12.7)	39.7 (9.5)	43.1 (13.2)
BMI, kg/m^2^, mean (SD)	25.9 (3.9)	27.1 (4.2)	24.2 (3.9)	24.1(3.8)
Sex, women, No. (%)	114 (71.2)	31 (83.8)	11 (55.0)	32 (66.7)
First presentation, yes, No. (%)				
Non-specific symptoms	40 (25.0%)	20 (54.1)	8 (40.0)	22 (45.8)
Asymptomatic	20 (12.5)	5 (13.5)	6 (30.0)	9 (18.8)
Acute hepatitis	58 (36.2)	7 (18.9)	2 (10.0)	10 (20.8)
Cirrhosis	52 (32.5)	6 (16.2)	5 (25.0)	9 (18.8)
Common comorbidities, yes, No. (%)				
Hypothyroidism	8 (5.0%)	2 (5.4%)	0 (0.0%)	1 (2.1%)
Crohn’s disease	1 (0.06%)	1 (2.7%)	0 (0.0%)	0 (0.0%)
SLE	2 (1.2%)	0 (0.0%)	0 (0.0%)	2 (4.2%)
UC	2 (1.2%)	0 (0.0%)	8 (40.0%)	5 (10.4%)
RA	7 (4.4%)	1 (2.7%)	2 (10.0%)	0 (0.0%)

AIH: autoimmune hepatitis; PBC: primary biliary cholangitis; PSC: primary sclerosing cholangitis; BMI: body mass index; SLE: systemic lupus erythematosus; UC: ulcerative colitis; RA: rheumatoid arthritis

The most common first main clinical presentations in patients were categorized into non-specific symptoms, asymptomatic, and acute hepatitis. Non-specific symptoms as another common first manifestation was recorded in 40 (25.0%) AIH, 20 (54.1%) PBC, 8 (40.0%) PSC, and 22 (45.8%) overlap syndrome patients. 20 (12.5%) patients with a diagnosis of AIH, 5 (13.5%) patients with a diagnosis of PBC, 6 (30.0%) patients with a diagnosis of PSC, and 9 (18.8%) patients with a diagnosis of overlap syndrome were asymptomatic and were diagnosed only based on laboratory and histologically results. Acute hepatitis was observed mainly in patients diagnosed with AIH compared to other groups of patients. 58 (36.2%) patients in AIH, 7 (18.9%) in PBC,2 (10.0%) in PSC, and 10 (20.8%) in overlap syndrome had an acute hepatitis in their first clinical presentation.

The most common comorbidities were ulcerative colitis (UC), hypothyroidism, and rheumatoid arthritis (RA) which UC was more frequent among PBC and overlap syndrome patients, and hypothyroidism and RA were more frequent among AIH patients. Other comorbidities such as Crohn’s disease and Systemic lupus erythematosus were also found in the study population which is represented in detail by groups in the table.

**Fig. 1:**
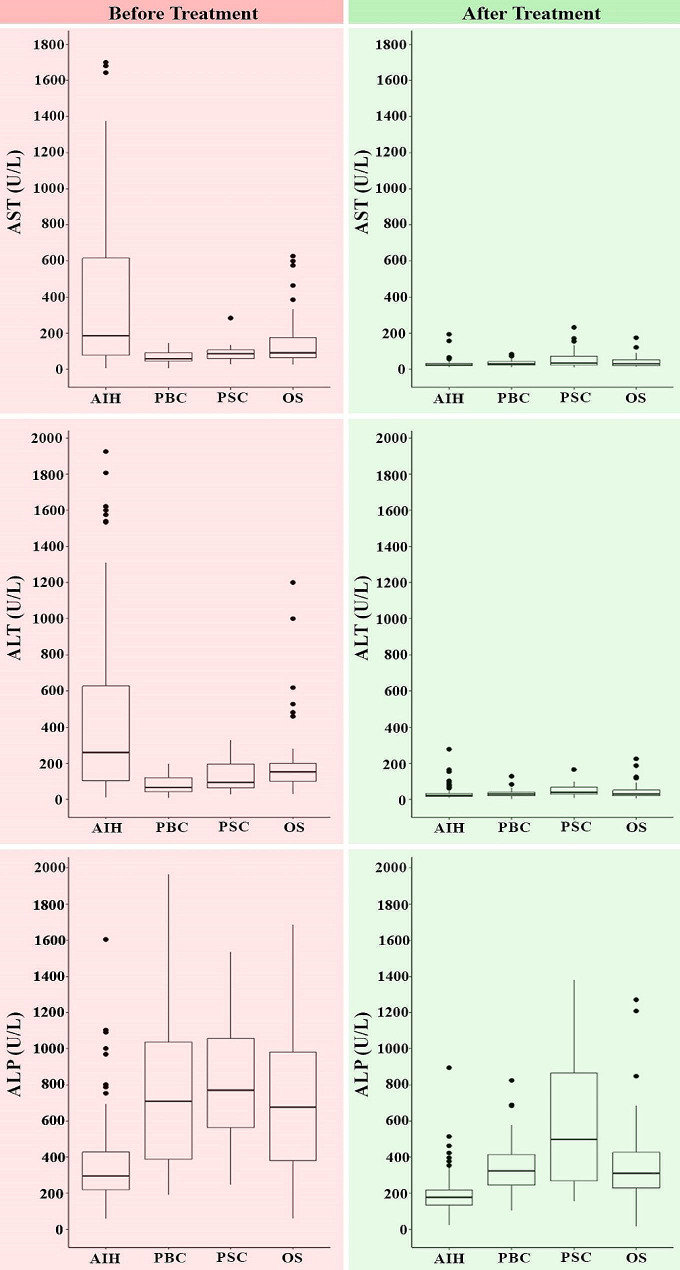
Biochemical levels of liver enzymes before and after treatment

Regarding laboratory parameters at the time of diagnosis, individuals diagnosed with AIH exhibited elevated transaminase levels. Conversely, those diagnosed with PBC and PSC displayed increased alkaline phosphatase levels in comparison to AIH patients. In the case of overlap syndrome patients, both transaminases and alkaline phosphatase were observed at high concentrations. Moreover, Liver enzyme levels were significantly diminished after treatment which is illustrated in Fig. 1.

Regarding laboratory variables, ANA, ASMA, AMA, anti LKM1, and anti-dsDNA were evaluated among patients’ population and presented in Table 2. ANA was positive most frequent in AIH patients with 67 (41.9%) participants. Moreover, 7 (18.9%) in PBC, 2 (10.0%) in PSC, and 13 (27.1%) in overlap syndrome also had positive ANA laboratory test. AIH and overlap syndrome patients had the most frequent ASMA-positive results with 21 (13.1%) and 11 (22.9%) participants, respectively. AMA was positive 25 (67.6%) PBC patients. 7 (4.4%) AIH and 7 (14.6%) overlap syndrome patients were also had AMA-positive results. Regarding other laboratory test such as anti-LKM1 and anti-dsDNA only AIH groups had positive results with 5 (3.1%) and 7 (4.4%) patients, respectively.

**Table 2:** Laboratory characteristics and biopsy sampling of study population

	AIH (n = 160)	PBC (n = 37)	PSC (n = 20)	Overlap syndrome (OS) (n = 48)
ANA	67 (41.9%)	7 (18.9%)	2 (10.0%)	13 (27.1%)
ASMA	21 (13.1%)	1 (2.7%)	3 (15.0%)	11 (22.9%)
AMA	7 (4.4%)	25 (67.6%)	1 (5.0%)	7 (14.6%)
Anti-LKM1	5 (3.1%)	0 (0.0%)	0 (0.0%)	0 (0.0%)
Anti-dsDNA	7 (4.4%)	0 (0.0%)	0 (0.0%)	0 (0.0%)
Liver Biopsy	121 (75.6%)	24 (64.8%)	12 (60%)	34 (70%)

AIH: autoimmune hepatitis; PBC: primary biliary cholangitis; PSC: primary sclerosing cholangitis ANA: antinuclear antibody; ASMA: anti−smooth muscle antibody; AMA: anti−mitochondrial antibody; Anti−LKM: anti−liver−kidney microsomal antibody; Anti−DSDNA: anti double−stranded deoxyribonucleic acid

**Fig. 2:**
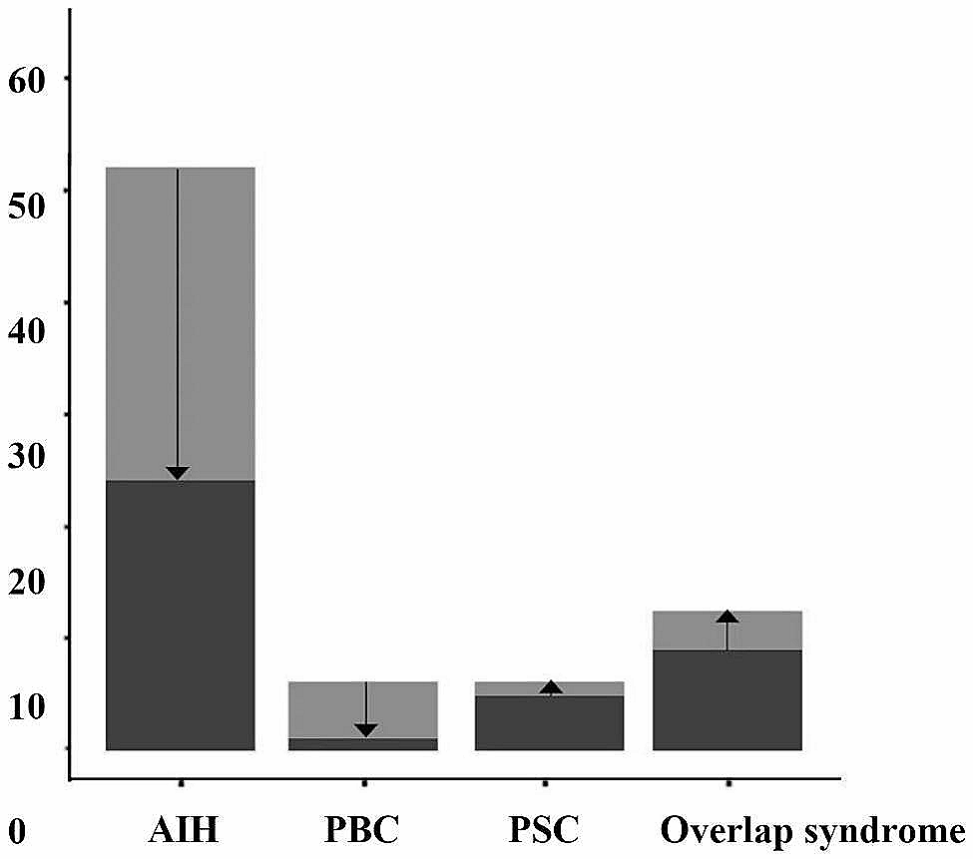
Cirrhosis status of patients at baseline and after treatment

With regard to cirrhosis, 52 (32.5%) patients with AIH were clinically, radiologically, biochemically, or histologically cirrhotic (in various stages) at the time of diagnosis; cirrhosis was also recorded as a main manifestation in 6 (16.2%) PBC, 5 (25.0%) PSC, and 9 (18.8%) overlap syndrome patients’ medical records. the AIH patient study started with 52 patients with cirrhosis and 28 (53.8%) of them showed regression from cirrhosis in response to treatment at the end of follow-up. Details in cirrhosis status at baseline and after treatment are presents in Fig. 2.

Drugs most commonly used for treatment populations of patients are summarized in Table 3. Prednisolone was administrated in 145 (90.1%) patients of AIH and 44 (91.7%) patients of overlap syndrome. Azathioprine was also most frequently used in AIH and overlap syndrome patients with 151 (94.4%) and 41 (85.4%) participants, respectively. Moreover, Azathioprine has been administrated for 6 (16.2%) PBC and 5 (25.0%) PSC patients. Ursodeoxycholic acid (UDCA) was another most frequently administrated drug among patients in their medical records. 63 (39.4%) AIH, 37 (100.0%) PBC, 20 (100.0%) PSC, and 48 (100.0%) overlap syndrome patients had a history of UDCA administration (Table 3).

**Table 3:** Study population treatment

	AIH (n = 160)	PBC (n = 37)	PSC (n = 20)	Overlap syndrome (n = 48)	
Azathioprine	151 (94.4%)	6 (16.2%)	5 (25.0%)	41 (85.4%)	
UDCA	63 (39.4%)	37 (100.0%)	20 (100.0%)	48 (100.0%)	
Prednisolone	145 (90.1%)	1 (2.7%)	1 (5.0%)	44 (91.7%)	
Mycophenolate Mofetil	18 (11.2%)	0 (0.0%)	0 (0.0%)	7 (14.6%)	
Cyclosporine	8 (5.0%)	0 (0.0%)	0 (0.0%)	2 (4.2%)	
Budesonide	5 (3.1%)	0 (0.0%)	0 (0.0%)	0 (0.0%)	

AIH: autoimmune hepatitis; PBC: primary biliary cholangitis; PSC: primary sclerosing cholangitis; UDCA: ursodeoxycholic acid

Regarding response to treatment among study population, complete response occurred in 112 (70%) of AIH and 28 (58.4%) of overlap syndrome patients. During follow-up time 21 patients were not respondents to administrated treatment. Of these participants, 11 (6.9%) had AIH and 10 (20.8%) had overlap syndrome. Insufficient response was also observed among 37 (23.1%) patients of AIH and 10 (20.8%) patients of overlap syndrome. For patients with overlap syndrome, 12 (25%) patients had cirrhosis at end of the follow-up period. Details in response to treatment evaluation and outcomes in AIH and overlap syndrome patients are shown Table 4.

**Table 4:** AIH and Overlap syndrome Study population response to treatment evaluation and outcomes

	AIH (n = 160)	Overlap syndrome (n = 48)
Non-response	11 (6.9%)	10 (20.8%)
Complete response	112 (70.0%)	28 (58.4%)
Insufficient response	37 (23.1%)	10 (20.8%)

AIH: autoimmune hepatitis

Moreover, during follow-up period of study, 32 patients form AIH participants have episodes of without medication. From these patients, during median period of 5.0 (IQR 3.5–24.0) months 12 patients experienced relapsing and also 20 of them during median period of 12.0 (IQR 6.0–24.0) months did not show relapse till end up study follow-up.

For PBC and PSC patients’ classification with respect to the response to UDCA treatment, 32 (91.9%) and 8 (40%) of PBC and PSC patients responded to UDCA, respectively. Non- responsive patients were 5 (8.1%) in the PBC group and 12 (60%) in the PSC group (Table 5).

**Table 5:** PBC and PSC Study population response to treatment UDCA

	PBC (n = 37)	PSC (n = 20)
Non-response	5 (8.1%)	12 (60.0%)
Response	32 (91.9%)	8 (40.0%)

PBC: primary biliary cholangitis; PSC: primary sclerosing cholangitis

All 5 non-responder patients in PBC were suffering from itching and fatigue (non-specific symptoms) as first presentation.

In terms of cirrhosis, while the study initially had 6 (16.5%) patients with cirrhosis in the PBC group, only 1 (2.7%) remained by the end of follow-up. In contrast, there was an increase from 5 (25%) to 6 (30.0%) cirrhotic patients in the PSC group.

## Discussion

This study is the one of the first to evaluate the clinical course, serologic profile, treatments, and complications of AILD in an Iranian population. Regarding the general insight about the patients during the follow-up, the patients experienced different evolutions during the follow-up period according to AILD subgroups, which will be discussed in detail below.

### AIH and overlap syndrome

Various studies reported different prevalence for AIH among AILD patients’ population, but commonly suggested a female predominance pattern with a mean age of approximately 50 years for AIH. Tanaka et al. evaluated AIH in the Asia-Pacific region and reported the largest epidemiologic study was conducted in Japan, with a mean age of 59.9 and 85.8% female predominance [9]. Shen et al. reported a 28.5% prevalence for AIH among AILD patients with a mean age of 57.47 years and 84.1% female patients [2]. Other studies revealed similar results with the mean age of diagnosis ranging 48.4 to 58 years old and a female predominance between 67.7 and 89.7% [7, 8, 20]. Our study, revealed an AIH prevalence of 60.4% with the mean age of the patients was 46.4 years, with a 71.2% female predominance in accordance with prior studies. The clinical age of onset of AIH exhibits significant heterogeneity. As observed in our study, the majority of previous studies have reported the onset of AIH in non-elderly populations. However, it is important to note that diagnoses of AIH in the elderly population have also been documented, but it is more frequently asymptomatic in this age group [21].

Overlap syndrome, similar to AIH, has a female predominance pattern with up to 100% female cases in some studies [3]. Lamba et al. reported a mean age of 55 years and a female predominance of 67% [7]. Al-chalabi et al. reported a median age of 54.5 for PBC/AIH overlap syndrome, with a female predominance of 80% [22]. In our study, 18.11% of the cases were regarded as overlap syndrome patients with a mean age of 43.1 years and a female predominance of 66.7% which is relatively younger than AIH patients and concurs with previous investigations.

The most common presentation among AIH patients in Diaz-Ramirez et al. study was acute hepatitis [20]. Furthermore, 83.8% positive serologic markers, including ASMA or ANCA, were reported, with an 81.8% positivity reported for AMA in overlap syndrome patients. Tanaka et al. reported ANA and ASMA elevations as common and occasional presence of anti-LKM1 as the characteristics of type 2 AIH [9]. Katsumi et al. reported that 46.4% of the patients with AIH present with acute hepatitis, 22.7% with cirrhosis, and 4.3% with signs of liver failure upon diagnosis with the positivity of ANA and ASMA as characteristic features for AIH due to hepatocellular injury [23]. In this study, the most common presentation in AIH was acute hepatitis, with a prevalence of 36.2%. Cirrhosis was seen in 32.5% of the AIH patients, and the most common presentation of overlap syndrome was non-specific presentations. The most common positive serologic marker in AIH patients was ANA. In this study, Anti-LKM1 and Anti-dsDNA were only positive in AIH patients. Furthermore, ANA was the most positive serologic marker in overlap syndrome patients with no Anti-LKM1 and Anti-dsDNA reports.

The slight difference in ANA in comparison with other reports [14] might be explained at the light of the laboratory tests (ELISA instead of immunofluorescence assays (IFA)) used for autoantibody detection.

Diaz-Ramirez et al., similar to Invernizzi et al. [3], and Floreani et al. [24], reported hypothyroidism as the most common comorbidity seen among 34.2% of the AILD patients [20]. Fernandez et al. reported systemic lupus erythematous to be the most common associated disease with 12.1%, while thyroid disease was seen in 4.8% of the patients [25]. In this study, among AIH patients, hypothyroidism was the most common comorbidity, with a prevalence of 5%, and was followed by RA. However, UC was the most commonly reported comorbidity in overlap syndrome with a prevalence of 10.4%.

Common regimens for AIH are monotherapy with prednisolone or combination therapy of prednisolone and azathioprine which are targeted at inflammation and fibrosis alleviation [26]. Diaz-Ramirez et al. used azathioprine and prednisolone as the main induction and maintenance therapy medication, and, similar to Floreani et al. [24], added UDCA for overlap syndrome patients [20], which resulted in a 84.9% biochemical remission and 12.2% partial remission in Diaz-Ramirez et al. study, and 82.19% remission in Floreani et al. study. Many studies suggest prednisolone alone or combined with azathioprine as the standard therapy for AIH, and overlap syndrome with complete response rates ranging from 79 to 96.1% for AIH, 62.5% for PBC/AIH overlap, and 87.5% for PSC/AIH overlap syndromes [3, 22, 25, 27]. Tanaka et al., evaluating 32 patients with PBC/AIH overlap syndrome, used corticosteroid with and without UDCA in 8, and 6 patients, respectively while using UDCA monotherapy in 15 patients, and achieved complete response in all patients [28]. In a study by Tamimi et al. 30 AIH patients were treated initially with prednisolone, with and without azathioprine [26]. Maintenance therapy was provided with a combination of prednisolone and azathioprine in 66.7%, with prednisolone as single therapy in 16.7% of cases. In 13.3% of cases that standard therapy failed or were intolerant to azathioprine, either mycophenolic acid or budesonide were administered for maintenance therapy which led to complete and partial biochemical remission in 66.7%, and 13.3% of the whole patients within 6 months, respectively. In the present study, the most common treatment regimen in AIH patients was azathioprine and prednisolone combination for induction therapy, which yielded 70% complete remission rate among AIH patients and 58.4% among overlap syndrome patients, and 23.1% partial remission rate among AIH, and 20.8% among overlap syndrome patients. More than 90% of cases with AIH and overlap syndrome, received corticosteroid for induction therapy. Moreover, Mycophenolate Mofetil, Cyclosporine, and Budesonide were only used in AIH patients, in non-responsive cases, and difficult-to-treat cases. UDCA was used more commonly in all overlap syndrome patients, while only 39.4% of AIH patient received UDCA in indefinite diagnosis cases early in the disease course. Moreover, Lack of response to medication was seen in 6.9% of the AIH, and 20.8% of the overlap syndrome patients. In general, combination therapy with corticosteroids and immunosuppressive therapy is preferred over monotherapy to reduce the dosage of corticosteroids. Moreover, tapering of corticosteroids is preferrable within 6 months after the initiation of the therapy to mitigate related side effects. The most common medication for maintenance therapy in AIH was azathioprine.

Natural course of AIH and overlap syndromes result in several complications. Diaz-Ramirez et al. reported cirrhosis and transplantation in 37.8% of the AIH patients, and 10.1% of all patients [20]. Puustinen et al. reported 2.5% transplantation among AIH patients in a nationwide registered study conducted in Finland [29]. In another study by Floreani et al. study, complications were seen in a total of 21 patients with 7 transplantations [24]. Al-Chalabi et al. reported 23.9%, 20%, and 43.8% total death or liver transplantation in AIH, PBC/AIH and PSC/AIH overlap syndromes, respectively. In Fernandez et al. study, cirrhosis was present in 39% of the patients at the beginning of the study, and during follow-ups, 34.1% of patients without cirrhosis, became cirrhotic among whom, 23.2% developed decompensation [25]. In this study, 52 cases (32.5%) of AIH, and 9 cases (18.8%) of overlap syndrome patients were diagnosed with cirrhosis in the beginning of the study, which signs of regression were seen as response to therapy at the end of the study in 28 cases (53.8%) of AIH patients; while 12 cases (25%) of cirrhosis was reported at the end of the study among overlap syndrome patients. Among 32 AIH patients who attempted discontinuation of the medication, relapse episodes occurred in 12 patients with a median period of 5 months, while the other 20 cases did not experience relapse episodes until end of study.

The impact of different genetic backgrounds has been shown to contribute to the diverse autoantibody and clinical profiles observed in autoimmune hepatitis (AIH) worldwide. Previous studies have demonstrated that variations in genetic factors play a significant role in shaping the presentation and progression of AIH. These genetic differences can influence the production of autoantibodies, such as antinuclear antibodies (ANA), smooth muscle antibodies (SMA), liver-kidney microsomal antibodies type 1 (LKM-1), and others. Additionally, these genetic variations may also affect the severity of liver inflammation and the response to treatment. Our study results also hint potential differences in the autoantibody/clinical profiles of AIH patients in Iran compared to other regions of the world, as well as variations in their responses to treatment. These differences highlight the need for further attention to the genetic background among the Iranian population in future studies involving AIH patients. Understanding the influence of genetic backgrounds is crucial for unraveling the complex nature of AIH and may provide insights into personalized approaches to diagnosis, treatment, and disease management tailored to specific populations.

### PBC and PSC

In Shen et al. study, 50.8% of the cases were PBC patients with a mean age of 56 years and a female proportion of 79.7% [2]. With the exclusion of overlap syndrome patients, Webb et al. reported a similar 47.88% PBC fraction among AILD patients. In Webb et al. study, 86.8% of patients were female, with a 6.6:1 female-to-male ratio and a mean age of 63 [8]. Lamba et al. reported a mean age of 57.9 years and a female predominance of 77% [7]. In the present study, PBC accounted for 14% of the patients with a mean age of 51.2 years and a female predominance of 83.8%, similar to previous studies. This difference in the prevalence of PBC among studies may be due to small sample sizes and differences in inclusion criteria for in this study all AILD cases were included while some studies excluded overlap syndrome cases. Fifty to 60% of patients suffering from PBC are asymptomatic and identified after screening tests. Overt symptoms develop in most patients within 2 to 4 years while some individuals are asymptomatic for years. In the present study, there are more patients with AIH than PBC which is rare. It is maybe the result of the impaired systematic screening test and medical records. Moreover, ethnic differences may also play a part in different prevalence of PBC and PSC which needs further investigation.

Invernizzi et al. reported PSC to be predominant among males [3]. Webb et al. reported a prevalence of 12.86% for PSC with a male predominance of 58.1% and a mean age of 57 years [8]. Lamba et al. also reported a mean age of 52 years, with 50% of them being female [7]. In the present study, PSC accounted for 7.5% of the patients with a mean age of 39.7 years and a female predominance of 55%, which does not agree with previous studies that reported a male predominance pattern for PSC; whoever, this can be due to few numbers of PSC patients in our study.

Hasegawa et al. reported PBC and PSC to present initially by asymptomatic liver dysfunctions [30]. Fatigue, reported in 36% of the patients, was the most common symptom among patients in Garioud et al. study, and cirrhosis was present in 16.5% at the time of enrolment [31]. Similarly, Fricker et al. reported asymptomatic liver elevations in up to 50% of cases PSC patients [32]. In this study similar to previous studies, PBC and PSC mostly had non-specific symptoms. Acute liver failure was seen in 18.9%, and 10.0% of PBC, and PSC cases; Moreover, cirrhosis was seen in 16.2%, and 25% cases, respectively.

Due to the high sensitivity and specificity of AMA, a high AMA accompanying a high Alkaline phosphatase level is strongly in favor of PBC diagnosis [33]. Peerani et al. reported 17% of PSC patients to be ASMA positive [34]. In this study, similar to Peerani et al. study, ASMA was positive in 15% of the PSC patients, and AMA was the most sensitive serologic marker in PBC patients which was positive in 67.6% of the cases. Studies have reported the importance of ANA and its staining pattern in PBC-specific ANA [35]. In the present study, 18.9% of PBC patients were ANA positive. Besides 13.8% of our patients were PBC-specific ANA. Regarding ELISA method, we used for ANA assay, we don’t have any data about the histological pattern.

Mago et al. reported Sjogren syndrome, thyroid dysfunction, and systemic sclerosis as PBC’s most associated underlying comorbidities [36]. Invernizzi et al. reported PBC, and PSC to be related to Sjogren’s syndrome, and Crohn’s disease, respectively [3]. Garioud et al. reported concomitant Crohn’s disease, ulcerative colitis, and indeterminate IBD in 27%, 59%, and 14% of the patients respectively [31]. In our study, the most common comorbidities were ulcerative colitis which was seen in 40% of the PSC patients. Rheumatoid arthritis was also seen in 10% of PSC patients. Similar to AIH, the most common comorbidity among PBC patients was hypothyroidism as well.

Better treatment response has been reported for PBC compared to PSC. Hasegawa et al. reported UDCA to significantly improve the conditions in patients with PBC [30]. Invernizzi et al. reported UDCA as one of the most important factors regarding response variability among PBC and PSC patients [3]. Trivedi et al. reported an 18.6 in 1000 person per year rate of cirrhosis among PSC cases [37]. In the present study, UDCA was the most used therapeutic option in PBC and PSC patients and led to 91.9%, and 40% remission in PBC, and PSC, respectively, which shows poor response to treatment in PSC patients.

All 5 non-responder patients in PBC were suffering from itching and fatigue (non-specific symptoms) as first presentation, which shows that patients who came with itching and fatigue symptoms had weaker response to treatment and more likely to get complications of PBC as other study show that [38].

Garioud et al. reported, 17% of the patients undergoing liver transplantation and the detection of new malignancies in 4% of the population [31]. In our study, cirrhosis was present in 6 (16.2%), and 5 (25%) PBC, and PSC cases, respectively which resulted in 1 (2.7%) PBC, and 6 (30%) PSC cases at the end of the study. Response to treatment was observed in PBC cases leading to regression of 5 cases (83.3%); while cirrhotic cases were increased in PSC cases (by 20%). This finding, in accordance with previous literature suggests an effective role for UDCA, as the main treatment choice for PBC, rather than PSC. Moreover, UDCA administration in PSC cases may result in laboratory resolution while the disease is progressing, and moving toward development of cirrhosis. Furthermore, in this study transplantation was seen in 5 (1.8%) cases.

The genetic background of each patient plays a crucial role in influencing the course of the disease. A comprehensive exploration of the genetic profile within the population holds the potential to enhance our understanding of the underlying mechanisms and improve outcome predictions. Notably, the established correlation between Human Leukocyte Antigen (HLA) markers and various forms of liver diseases underscores the contribution of genetics to the diverse manifestations observed across different geographic locations and populations [39, 40].

In our investigation as the Iranian population, 3% of cases were Anti-LKM1-positive. Nevertheless, comprehensive records regarding the prevalence of Anti-LKM1 positivity among AILD patients are currently lacking. This underscores the need for additional research to fill this gap in knowledge.

Although, in a general view, the findings of this study are in agreement with the global results published from Europe and the USA, however, there are differences due to race, lifestyle, diet, medication compliance, missing in the medical record system, difference in laboratory methods, and routine screening program were the main limitations of this study, which provided the basis for the mismatch of findings in some cases.

This is one of the first studies to evaluate the clinical course and complications in an Iranian population; however, this study has limitations. Small sample size, single-center and incomplete data are limitations of this study. Further studies with more comprehensive sample sizes, and patients’ follow-ups more commonly would yield more and useful information. As another limitation in our study, we utilized the simplified scoring system exclusively for evaluating our patients. It is important to note that both proposed scoring systems are not interchangeable and each has its utility in specific clinical situations. The original scoring system holds greater value in diagnosing patients with few or atypical features of AIH, particularly those with cryptogenic or autoantibody-negative chronic hepatitis. On the other hand, the simplified scoring system is more useful for excluding the diagnosis in patients with distinct etiologically-driven diseases who also present with immune manifestations.

## Conclusion

In conclusion, among Iranian population, non-specific symptoms such as fatigue and itching were the most frequent first main presentations for AILD. Proper administration of medications in AILD patients lead to regression from cirrhosis and improvement of manifestations; while, discontinuation of medication may cause relapses. In total, AILD patients are mostly responsive to medications and their complications can be controlled except for PSC cases who show limited response.

## Data Availability

The datasets used and/or analyzed during the current study available from the corresponding author on reasonable request.
